# Mass and stiffness sensing performance of nanomechanical resonators: viability of infectious virus detection

**DOI:** 10.1186/s11671-025-04295-7

**Published:** 2025-07-10

**Authors:** Manuel Gómez-Moreno, Juan Molina, José J. Ruz, Óscar Malvar, Javier Tamayo, Montserrat Calleja, Álvaro San Paulo

**Affiliations:** Instituto de Micro y Nanotecnología, IMN-CNM, CSIC (CEI UAM+CSIC), Isaac Newton 8, E-28760 Tres Cantos, Madrid, Spain

## Abstract

We examine the performance of nanomechanical resonators for mass and stiffness sensing of nanoparticulate analytes with focus on their application for untargeted infectious virus detection. The characteristic narrow mass distributions of viruses, together with the existing correlations between their stiffness and infectivity, point out to nanomechanical sensors as a particularly suited alternative to molecular detection techniques, constrained by limited processing speed, target-specificity, and the inability to directly assess infectivity. We present a theoretical analysis of the response of flexural beam resonators to the adsorption of nanoparticulate analytes, and derive analytical expressions for the mass and stiffness sensing responsivity, resolution and signal to noise ratio as a function of the beam characteristics and analyte adsorption parameters. We demonstrate that both the mass and stiffness of viruses can contribute to resonance frequency shifts that significantly exceed the fundamental detection limits of beams with plausible dimensions and for realistic adsorption parameters. Particularly, stiffness resolution can reach levels well below the stiffness variations observed in some viruses as a consequence of maturation, enabling an integrated approach for infectivity assessment. We conclude that the practical application of nanomechanical spectrometry for infectious virus detection is not limited by the performance of state-of-the-art sensor technology, but by the efficiency of analyte delivery methods, encouraging future research on optimizing their implementation.

**Supplementary Information:**

The online version contains supplementary material available at 10.1186/s11671-025-04295-7.

## Introduction

SARS-CoV-2 has made evident our lack of sufficiently efficient technologies for preventing, containing and mitigating the effects of virus transmission during epidemic and pandemic outbreaks [1]. In particular, currently established virus detection methods for either infection diagnosis or environmental monitoring present functional limitations that strongly compromise our response to pandemic crises as the one recently experienced with the COVID-19 disease [2, 3]. Selective sensing approaches directed at predetermined molecular targets have been mostly adopted in the pursue of fast detection methods [4]. The three main established technologies are: (i) identification of viral gene regions by nucleic acid detection techniques, such as polymerase chain reaction (PCR) technologies, gene sequencing or CRISPR-based detection; (ii) recognition of antibodies produced in response to viral infection (IgM and IgG); and (iii) detection of antigens (spike, envelope, or nucleotide proteins) by lateral flow assays or ELISA technologies. PCR based methods represent the gold standard for virus detection, as in general they ensure high accuracy, sensitivity and specificity. Yet, this approach requires sophisticated equipment and it is hindered by the slow delivery of the results after sampling (from several hours to a few days). Serological tests rely on simpler technologies, but are limited to human samples where low concentrations of the targeted analyte decrease their reliability. Similarly, methods based on antigen detection can provide results in minutes, but require relatively large viral loads in order to minimize the levels of false positives and negatives. All these molecular sensing techniques are disease-specific and their adaptation to new viruses or mutations requires significant effort and time. In addition, they share one particularly important limitation, which concerns their inability to provide information on virus viability. To date, the only way to obtain information on the viability of a virus sample is by cell culture, which can take up to weeks depending on host cell and virus type.

The urgent demand for fast, untargeted and viability-sensitive virus detection has precipitated a rapidly increasing research on alternative sensing technologies [5]. An approach radically different to those relying on biochemical detection is based on harnessing the unique mechanical properties of viruses, in particular, their mass and stiffness. The mass of most virus types known to spread by airborne transmission, such as measles virus, influenza virus, respiratory syncytial virus (RSV), human rhinovirus (hRV), adenovirus or coronavirus, lays in the 0.01 to 100 fg range [3, 6, 7]. But most significantly, some viruses have very narrow size distributions (down to below 1% in FWHM) [8, 9], so that their mass offers a usable physical marker for their identification. On the other hand, virus stiffness provides a potential approach for infectivity assessment. Some viruses need to complete a maturation process after budding from host cells that implies internal reorganization in order to become infective. Such maturation process has been shown to correlate to significant changes in the stiffness of the viral particles, associated to variations in their apparent Young’s modulus [10–13]. The stiffness of various types of viruses has been intensively studied by atomic force microscopy, and the order of magnitude of the Young’s modulus found for most of them is around 1 GPa [14]. Observed variations in Young’s modulus as a consequence of maturation reach the order of a few hundred MPa [10–12].

Nanomechanical spectrometry (NMS) is an emerging technology that aims at detecting, identifying and characterizing nanoparticulate analytes by means of their distinctive mechanical properties, particularly their mass and stiffness [15–17]. NMS relies on nanomechanical sensors, typically beam resonators, which provide a measure of the mass and stiffness of individual analytes accreting on their surface by harnessing adsorption-induced resonance frequency shifts of their flexural modes. The added mass of adsorbed analytes produces a downshift in the resonator’s resonance frequencies, whereas their elastic deformation as a consequence of the resonator vibration introduces an upshift contribution proportional to their stiffness. Theoretical approaches that account both mass and stiffness contributions have been developed for various sensor and analyte configurations [18–20], highlighting the importance of stiffness effects particularly when the analyte-to-resonator mass ratio is very low [21, 22]. The experimental analysis of both mass and stiffness of nanoparticulate analytes from experimental tracking of flexural resonators has also been demonstrated [23]. In the case of viruses, the application of NMS has recently begun to be explored [24–26]. However, neither the sensors nor the experimental methodologies applied up to now were conceived to optimize stiffness measurements, so that sensing performance optimization has been limited to mass measurements [27]. As a consequence, the simultaneous measurement of mass and stiffness of virus particles has not been achieved yet. In fact, although stiffness measurements of viruses have been specifically modelled theoretically [24], the analysis of the resonator parameters and adsorption conditions that leads to simultaneously optimized mass and stiffness sensing in consideration of frequency noise remains to be addressed, not only for virus particles in particular, but for nanoparticulate analytes in general.

In this work, we analyze mass and stiffness sensing with flexural beam nanomechanical resonators applied for the characterization of spherical solid nanoparticulate analytes, with particular focus on the development of an untargeted virus detection approach with assessment of infectivity. Firstly, we present a revision of the theoretical framework that describes mass and stiffness sensitivity of nanomechanical resonators based on flexural cantilever beams. Our approach is based on previous studies that describe the overall sensing mechanism and the behavior of resonance frequency shifts of flexural resonators as a consequence of analyte adsorption [20, 23]: here, we introduce a stiffness parameter that allows a simplified and more intuitive description of stiffness-induced resonance frequency shifts. We particularize our analysis for cantilever beams (single clamp), as they offer a slight advantage in terms of dynamic range as compared to double clamp beams for the same dimensions and experimental conditions [28]. Secondly, we apply such theoretical approach for the analysis of sensing performance, deriving analytical expressions to describe mass and stiffness responsivity, resolution and signal to noise ratio (SNR) as a function of the sensor and analyte parameters. We particularize our analysis for analyte parameters consistent with virus SARS-CoV-2 under the assumption of thermomechanical noise-limited frequency stability. Furthermore, we derive design rules for sensor optimization, and conclude that flexural beams with dimensions that can be considered as standard from the perspective of established microfabrication and transduction technologies, are expected to provide a suitable performance for mass and stiffness sensing of virus particles in regard of all the sensing parameters considered. We finally discuss the requirements that such sensors imply for future improvements in the efficiency of experimental methods for delivering the analytes to the sensitive surface area of the sensors, enabling a new technology line towards rapid, untargeted and infectivity-sensitive virus detection founded on nanomechanical sensing.

## Results and discussion

### Equation of sensitivity

**Fig. 1:**
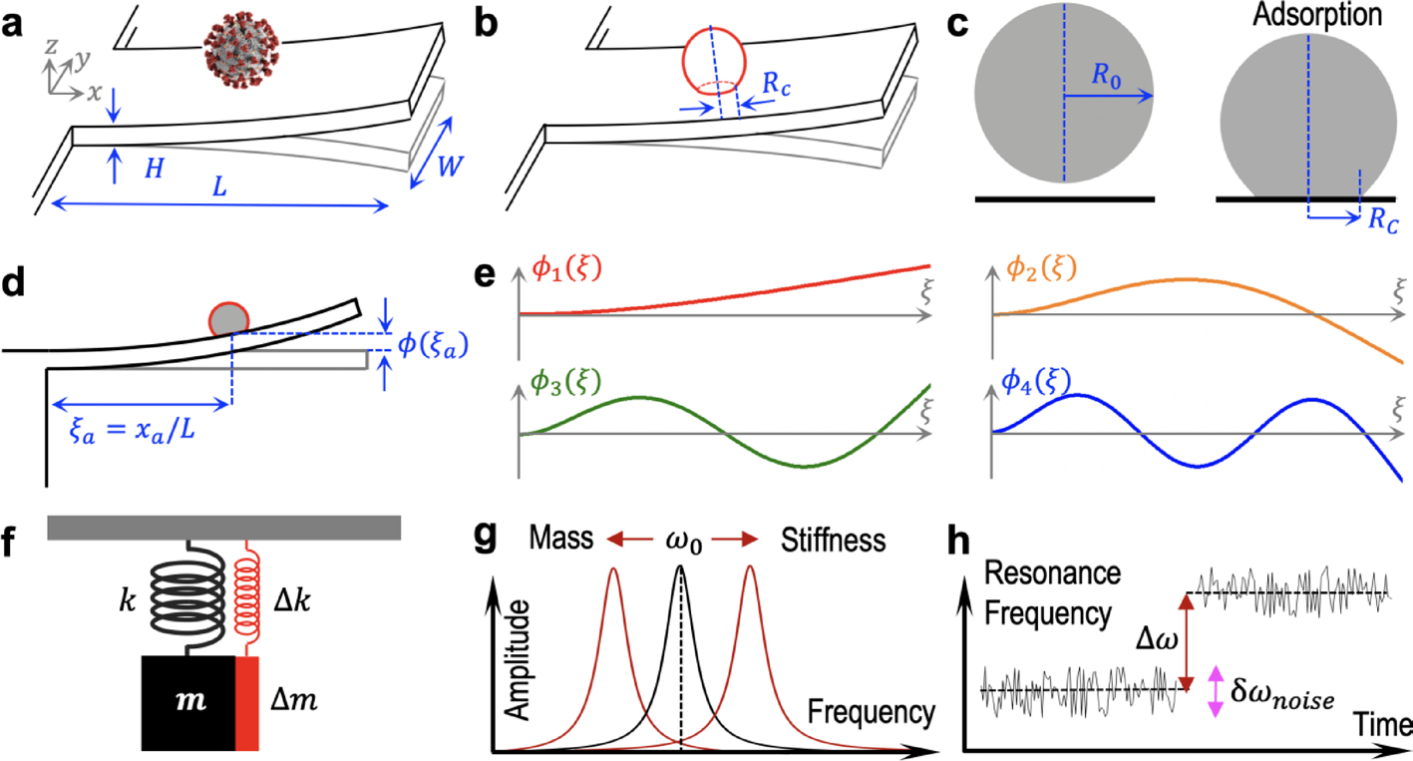
Principles of virus detection by mass and stiffness sensing with flexural beam nanomechanical resonators. **a** Schematic of virus adsorbed on a cantilever beam resonator, **b** Model of the adsorbed virus as a solid spherical nanoparticulate, **c** Numerical simulation of adsorption of a solid elastic nanosphere subject to Van der Waals forces, **d** Schematic of flexural beam vibration with the analyte adsorbed at a given normalized position ${\xi}_{a}$ where the n^th^ flexural mode shape is given by ${\varphi}_{n}\left({\xi}_{a}\right)$, **e** Mode shape of the first four flexural modes of a cantilever beam, **f** Equivalent point-mass one-dimensional spring model, **g** Schematic of resonance frequency shift generated by mass loading or stiffness coupling, **h** Schematic of resonance frequency shift generated by analyte adsorption compared with frequency noise

In this section we derive a simplified and meaningful expression that relates the mass and stiffness of nanoparticulate analytes adsorbed on cantilever beam resonators with the resonance frequency shift produced in their flexural modes as a consequence of analyte adsorption. We consider a singly clamped beam (i.e., a cantilever beam) with length $L$, width $W$, thickness $H$ and volume ${V}_{c}=LWH$ that satisfies $L\gg\, W,H$. A solid nanoparticulate analyte with spherical geometry and radius ${R}_{0}$ is assumed to deform at constant volume ${V}_{a}$ upon adsorption resulting in a circular contact area of radius ${R}_{c}$ (Fig. 1). We have performed finite element modeling (FEM) to estimate the resulting shape of such analyte upon adsorption when subject to Van der Waals interaction forces with the surface (Section S1, Supplementary Information). This shape will be assumed for all calculations presented in this work. The flexural displacement of the cantilever upon driven oscillation in the linear regime, $w$, can be derived from Euler-Bernoulli elastic beam theory [29], and it can be expressed as a sum of the contributions of n^th^ order eigenmodes as:

1 $$w\left(\xi,t\right)=\sum_{n\in\mathbb{N}}{A}_{n}{\varphi}_{n}\left(\xi\right)\text{cos}\left({\omega}_{n}t\right)$$

where $\xi=x/L$ is the normalized coordinate along the longitudinal axis. For each eigenmode of order $n$, ${A}_{n}$ is the oscillation amplitude, ${\varphi}_{n}\left(\xi\right)$ is the mode shape profile and ${\omega}_{n}$ is the angular resonance frequency. The mode shape profile plays a crucial role on sensing performance, and it is given by:

2 $${\varphi}_{n}\left(\xi\right)=\text{cosh}\left({\beta}_{n}\xi\right)-\text{cos}\left({\beta}_{n}\xi\right)+\frac{\text{cos}\left({\beta}_{n}\right)+\text{cosh}\left({\beta}_{n}\right)}{\text{sin}\left({\beta}_{n}\right)+\text{sinh}\left({\beta}_{n}\right)}\left[\text{sin}\left({\beta}_{n}\xi\right)-\text{sinh}\left({\beta}_{n}\xi\right)\right]$$

where ${\beta}_{n}$ satisfies $\text{cosh}\left({\beta}_{n}\right)\text{cos}\left({\beta}_{n}\right)+1=0.$ Figure 1e represents the mode shape of the first four flexural modes. The angular resonance frequency is given by:

3 $${\omega}_{n}^{2}=\frac{{\beta}_{n}^{4}}{12}\frac{{E}_{c}{H}^{2}}{{{\rho}_{c}L}^{4}}$$

where ${E}_{c}$ is the Young’s modulus and ${\rho}_{c}$ is the density of the cantilever material.

The resonant behavior of each flexural mode is analogous to that of a one-dimensional point-mass linear spring. The resonance frequency ${\omega}_{0}$ of a spring is determined by its mass $m$ and spring constant $k$ according to ${\omega}_{0}^{2}=k/m$. In consequence, small changes in the resonance frequency $\Delta{\omega}_{0}$ of a spring produced by small changes in its mass $\Delta m$ or spring constant $\Delta k$ can be expressed as:

4 $$\frac{\Delta{\omega}_{0}}{{\omega}_{0}}\approx\frac{1}{2}\frac{\Delta k}{k}-\frac{1}{2}\frac{\Delta m}{m}$$

This equation represents an intuitive view of the general sensing mechanism behind nanomechanical spectrometry: the effect of the adsorption of an analyte on the resonant response of the flexural modes of a beam can be both changing their effective mass by a small increment $\Delta m$ and/or changing their effective spring constant by $\Delta k$ (Fig. 1f). Thus, $\Delta m$ can be identified with an effective mass addition of the analyte, and $\Delta k$ with an effective contribution to the spring constant of the mode. We refer to these magnitudes as “effective” because the actual mass and stiffness of the analyte cannot be directly identified as the changes in mass and spring constant induced on each of the flexural modes upon analyte adsorption.

In order to compute the effective contributions of the mass and stiffness of the analyte upon adsorption on the beam to the resonance shift of its flexural modes,$\Delta{\omega}_{n}$, and most significantly, to relate them to characteristic properties of the analyte, it is useful to express the resonance frequencies of the flexural modes in terms of their maximum potential and kinetic energies per cycle, ${U}_{n}$ and ${T}_{n}$, respectively. This can be done by application of the Rayleigh-Ritz method [30], which, under the assumption that analyte adsorption produces a small change in potential and/or kinetic energy, results in the following expression (Section S2, Supplementary Information):

5 $$\frac{\Delta{\omega}_{n}}{{\omega}_{n}}=\frac{1}{2}\frac{{U}_{a,n}}{{U}_{n}}-\frac{1}{2}\frac{{T}_{a,n}}{{T}_{n}}$$

where ${U}_{a,n}$ and ${T}_{a,n}$are respectively the changes in potential and kinetic energy of the n^th^ mode produced as a consequence of analyte adsorption. The calculation of ${T}_{n}$ and ${T}_{a,n}$ can be performed by integrating, respectively over the corresponding cantilever and analyte volumes, the kinetic energy $dT$ of an infinitesimal volume $dV$ given by $dT=\frac{1}{2}\rho{\dot{w}(\xi,t)}^{2}dV$, where the dot denotes a time derivative. This results in:

6 $${T}_{n}=\frac{1}{2}{{\omega}_{n}^{2}M}_{c}{A}_{n}^{2}\int_{0}^{1}{\varphi}_{n}{\left(\xi\right)}^{2}d\xi$$

7 $${T}_{a,n}=\frac{1}{2}{{\omega}_{n}^{2}M}_{a}{A}_{n}^{2}{\varphi}_{n}{\left({\xi}_{a}\right)}^{2}$$

where ${M}_{c}={\rho}_{c}{V}_{c}$ and ${M}_{a}={\rho}_{a}{V}_{a}$ are the total inertial masses of the cantilever and analyte, respectively. The above equation for ${T}_{a,n}$ asumes that the volume of the analyte is much smaller than that of the cantilever, so that ${\varphi}_{n}\left(\xi\right)$ is approximately constant for an extension corresponding to the size of the analyte, adsorbed at position ${\xi}_{a}$. A similar calculation can be done for estimating ${U}_{n}$ and ${U}_{a,n}$ from the elastic potential energy corresponding to an infinitesimal volume, which is given by $dU=\frac{1}{2}{\sigma}_{ij}{\epsilon}_{ij}dV$, where ${\sigma}_{ij}$ and ${\epsilon}_{ij}$ represent the stress and strain tensor components within the volume [31]. The potential energy of the cantilever can be written as (Section S3.1, Supplementary Information):

8 $${U}_{n}=\frac{1}{2}\frac{{E}_{c}{V}_{c}}{12}\frac{{H}^{2}}{{L}^{4}}{A}_{n}^{2}\int_{0}^{1}{\varphi}_{n}^{\prime\prime}{\left(\xi\right)}^{2}d\xi$$

where double apostrophe denotes second derivative with respect to $\xi$. On the other hand, the strain distribution within the analyte can be quite complex, and it is not possible to make any assumptions a priori about such distribution that simplifies the calculation of ${U}_{a,n}$. However, it can be considered that the strain inside the adsorbate is a consequence of the transference of strain from the cantilever, so that the components of the strain in the analyte can be expressed as a linear combination of the in-plane components ${\epsilon}_{xx}^{c}$ and ${\epsilon}_{yy}^{c}$ of the strain of the cantilever at the point of adsorption [20], As a result, the potential energy contribution of the adsorbate to the n^th^ flexural mode can be expressed as (Section S3.2, Supplementary Information):

9 $${U}_{a,n}=\frac{1}{2}\frac{{E}_{a}{V}_{a}}{4}\frac{{H}^{2}}{{L}^{4}}\gamma{{A}_{n}^{2}\varphi}_{n}^{\prime\prime}{\left({\xi}_{a}\right)}^{2}$$

where ${E}_{a}$ is the Young’s modulus of the analyte. The dimensionless factor $\gamma$ carries the information about how well the components of the strain in the beam generate the same or other components in the adsorbed analyte, and thus it can be understood as a strain transference function.

From the above analysis we obtain the following expression for the frequency shift of flexural modes in terms of characteristic bulk properties of the cantilever and the analyte:

10 $$\frac{\Delta{\omega}_{n}}{{\omega}_{n}}=\frac{{3\gamma E}_{a}{V}_{a}}{{2E}_{c}{V}_{c}}\frac{{\varphi}_{n}^{\prime\prime}{\left({\xi}_{a}\right)}^{2}}{{\int}_{0}^{1}{\varphi}_{n}^{\prime\prime}{\left(\xi\right)}^{2}d\xi}-\frac{{M}_{a}}{{2M}_{c}}\frac{{\varphi}_{n}{\left({\xi}_{a}\right)}^{2}}{{\int}_{0}^{1}{\varphi}_{n}{\left(\xi\right)}^{2}d\xi}$$

Similarly to the expression for the one-dimensional point-mass spring (Eq. 4), we obtain a first term related to an effective stiffness contribution of the adsorbate, and a second term corresponding to an added effective mass effect. Following the analogy, we introduce a stiffness parameter $K$ for the cantilever and for the adsorbed analyte, which are respectively given by:

11 $${K}_{c}={E}_{c}{V}_{c}$$

12 $${K}_{a}={3\gamma E}_{a}{V}_{a}$$

The definition of this stiffness parameter $K$ from the product of Young’s modulus and volume is analogous to that of mass as the product of density and volume. The parameter $K$ has units of energy, and, for the cantilever, it is related to the effective spring constant ${k}_{n}$ of each flexural mode by ${k}_{n}=\frac{1}{12}{K}_{c}\frac{{H}^{2}}{{L}^{4}}{\int}_{0}^{1}{\varphi}_{n}^{\prime\prime}{\left(\xi\right)}^{2}d\xi$. We also define two coefficients ${c}_{K,n}\left({\xi}_{a}\right)=\frac{{\varphi}_{n}^{\prime\prime}{\left({\xi}_{a}\right)}^{2}}{{\int}_{0}^{1}{\varphi}_{n}^{\prime\prime}{\left(\xi\right)}^{2}d\xi}$ and ${c}_{M,n}\left({\xi}_{a}\right)=\frac{{\varphi}_{n}{\left({\xi}_{a}\right)}^{2}}{{\int}_{0}^{1}{\varphi}_{n}{\left(\xi\right)}^{2}d\xi}$ that contain the adsorption position dependence of the stiffness and mass terms, respectively, to find a compact expression for the sensitivity equation:

13 $$\frac{\Delta{\omega}_{n}}{{\omega}_{n}}=\frac{{K}_{a}}{{2K}_{c}}{c}_{K,n}\left({\xi}_{a}\right)-\frac{{M}_{a}}{{2M}_{c}}{c}_{M,n}\left({\xi}_{a}\right)$$

This equation makes evident that given a set of measurements of the frequency shift of a number of eigenmodes upon a particular analyte adsorption, the magnitudes of the analyte that can be directly inferred are its mass ${M}_{a}$ and its stiffness parameter ${K}_{a}$. It is important to note that whereas ${M}_{a}$ depends only on analyte properties, the effective stiffness of the analyte ${K}_{a}$ does not, as it changes also with the elastic coupling with the cantilever, which is quantified in the factor $\gamma$. As strain transference happens within the contact area between the analyte and the cantilever, it can be expected that $\gamma$ has a strong dependence on the contact radius ${R}_{c}$, which in experimental situations will be influenced by the actual adhesion and short-range forces between analyte and surface. Unfortunately, an exact analytical determination of $\gamma$ is not possible in general because of the complex strain distribution within the analyte and the arbitrariness of predetermining a contact area. However, if the adsorption of a given analyte results in statistically consistent values of $\gamma$, then the measurement of ${K}_{a}$ can be directly used for the purpose of characterizing, detecting and identifying the effective stiffness of the analyte.

### Stiffness of adsorbed nanoparticulate analytes

The effect of the analyte stiffness on the resonance shift of the cantilever flexural modes is determined by the effective stiffness parameter ${K}_{a}={3\gamma }_{a}{V}_{a}$. The factor $\gamma$ depends on how the strain transferred to the analyte from the cantilever surface is distributed within the analyte. In spite of the simple distribution of strain at the cantilever surface, the resulting strain distribution within the analyte is in general quite complex, preventing an exact analytical derivation of $\gamma$. We have performed FEM simulations in order to compute numerically the strain distribution within the analyte and to determine the behavior of $\gamma$ with the contact radius. Details about the FEM methodology are provided in Section S4 of the Supplementary Information.

**Fig. 2:**
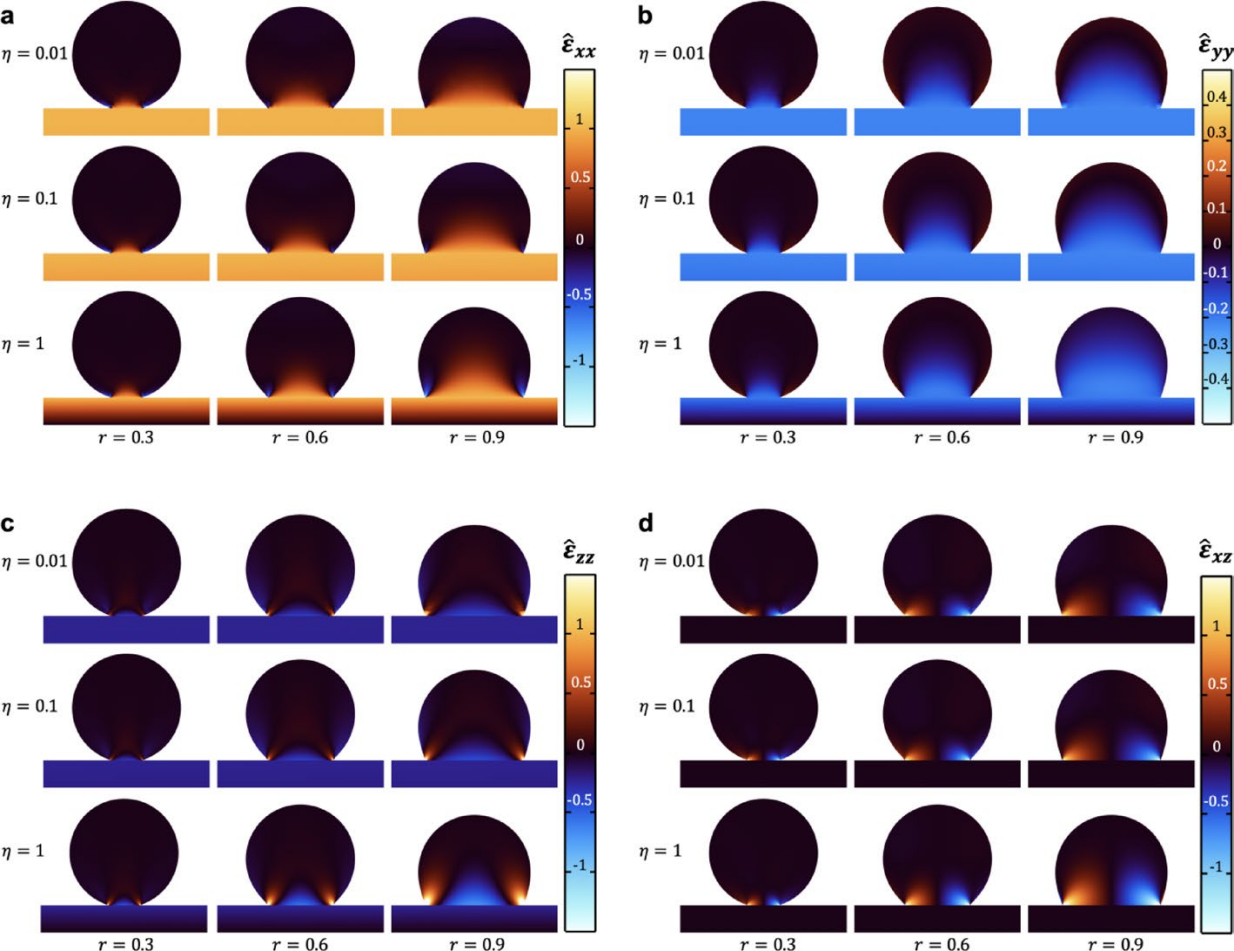
Finite element modeling of normalized strain components within an adsorbed analyte for different values of the ratio of analyte radius to beam thickness ${\eta=R}_{0}/H$, and relative contact radius ${r=R}_{c}/{R}_{0}$. **a** ${\epsilon}_{xx}$component, **b**${\epsilon}_{yy}$component, **c** ${\epsilon}_{zz}$component, **d** ${\epsilon}_{xz}$component

Figure 2 presents FEM calculations of the distribution of the ${\epsilon}_{xx}$,${\epsilon}_{yy}$, ${\epsilon}_{zz}$ and ${\epsilon}_{xz}$ strain components within an adsorbed spherical analyte for varying relative contact radius ${r=R}_{c}/{R}_{0}$ and relative analyte size to beam thickness ${\eta=R}_{0}/H$ (all other components are zero). In these calculations, the analyte has a Young’s modulus of 1 GPa and a Poisson’s ratio of 0.25. In all cases, each strain component is normalized to the ${\epsilon}_{xx}$component at the surface of the beam at the point of adsorption, ${\widehat{\epsilon}}_{ij}\equiv{\epsilon}_{ij}/{\epsilon}_{xx}^{c}({x}_{0},{y}_{0},{z}_{0})$. The represented normalized strain maps correspond to cross-sections of the analyte adsorbed on the cantilever surface along the longitudinal cantilever direction, $x$. The intricate behavior of the strain within the analyte is evident. However, we can distinguish between two different general trends for the absolute value of all strain components. The first one is a monotonic decrease along $z$ so that the strain goes to zero at the free surface of the analyte, which we identify as strain release. This behavior is dominant for the in-plane components ${\epsilon}_{xx}$ and ${\epsilon}_{yy}$ and it is also observed for ${\epsilon}_{zz}$ at centered positions. The other behavior is a non-monotonic trend with $z$ in which the strain within the analyte presents a maximum close to the cantilever surface, being this maximum larger than any value within the beam. This trend dominates in ${\epsilon}_{xz}$ and it is also observed for ${\epsilon}_{zz}$ and ${\epsilon}_{xx}$ prevailing at positions closer to the contact line, which relates it to edge effects. The strain release behavior is dominant at central positions for the in-plane strain components ${\epsilon}_{xx}$ and ${\epsilon}_{yy}$, whereas edge effects become more important in the out-of-plane components ${\epsilon}_{zz}$ and ${\epsilon}_{xz}$, especially for increasing contact radius and decreasing cantilever thickness.

**Fig. 3:**
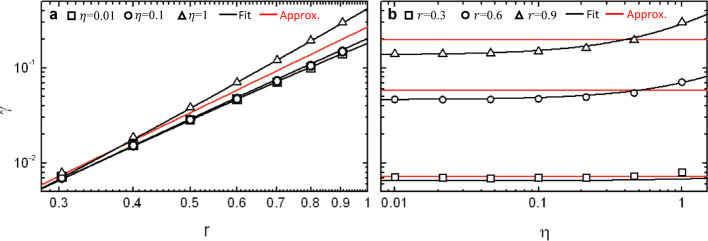
Calculation of the strain transfer function $\gamma$. **a** $\gamma$ as a function of $r$ for different values of $\eta$, **b** $\gamma$ as a function of $\eta$ for different values of $r$

FEM calculations also allow to examine the dependence of $\gamma$ on the contact radius and cantilever thickness (Section S5, Supplementary Information). The results are plotted in Fig. 3 with symbols, which shows $\gamma$ versus $r$ for different values of $\eta$. As expected, $\gamma$ presents a strong dependence on the contact radius, very close to ${\propto r}^{3}$, with an increasing contribution of $\eta$ for increasing $r$ (Fig. 3a). Remarkably, for $\eta<0.1$, the effect of $\eta$ is almost negligible for all values of $r$, which is evident when plotting $\gamma$ versus $\eta$ for fixed $r$ (Fig. 3b). In order to obtain an analytical approximation for $\gamma$, we fit the FEM calculations to:

14 $$\gamma\approx{\alpha}_{0}{r}^{3}\left(1+{\alpha}_{1}\eta+{\alpha}_{2}r+{\alpha}_{3}\eta r\right)$$

and obtain a remarkably good agreement for ${\alpha}_{0}=0.2711$, ${\alpha}_{1}=-0.3365$, ${\alpha}_{2}=-0.3366$ and ${\alpha}_{3}=1.2327$, as represented in Fig. 3 with black lines. This result implies that for sufficiently low values of $r$ and $\eta$, we can approximate $\gamma\approx{\alpha}_{0}{r}^{3}$, as represented in Fig. 3 with red lines. Under this approximation, the effective stiffness parameter is simplified to:

15 $${K}_{a}\approx{4\pi{\alpha}_{0}E}_{a}{R}_{C}^{3}\approx3.41{E}_{a}{R}_{C}^{3}$$

This approximation makes it possible to have a simple expression for ${K}_{a}$ which substantiates the dominant effect of the contact radius on the effective stiffness contribution of the analyte to the resonance frequency shift of the flexural modes. Thus, the requirement for statistical consistency of $\gamma$ in order to obtain meaningful estimations of ${K}_{a}$ in terms of analyte detection, translates directly to ${R}_{C}$. In other words, if variations in contact radius are sufficiently small, then variations in resonance frequency shifts arising from stiffness can be attributed to changes in the Young’s modulus of the analytes. Under these conditions, nanomechanical resonators provide a useful approach to measure the Young’s modulus distributions of populations of nanoparticulate analytes, a parameter which is otherwise difficult to characterize combining single-particle resolution and high-throughput.

### Responsivity

The responsivity of nanomechanical sensors to mass measurements is defined as the ratio of the resonance frequency shift observed in the sensor to the analyte’s mass, as given by ${\mathcal{R}}_{M,n}=\left|\partial{\omega}_{n}/\partial{M}_{a}\right|$ [32]. Thus, we can analogously introduce the responsivity to stiffness measurements as ${\mathcal{R}}_{K,n}=\left|\partial{\omega}_{n}/\partial{K}_{a}\right|$, so that the total resonance frequency shift produced in the n^th^ eigenmode is written as:

16 $$\Delta{\omega}_{n}={\mathcal{R}}_{K,n}{K}_{a}-{\mathcal{R}}_{M,n}{M}_{a}$$

where

17 $${\mathcal{R}}_{M,n}=\frac{{\omega}_{n}}{{2M}_{c}}{c}_{M,n}\left({\xi}_{a}\right)$$

18 $${\mathcal{R}}_{K,n}=\frac{{\omega}_{n}}{{2K}_{c}}{c}_{K,n}\left({\xi}_{a}\right)$$

Both mass and stiffness responsivities scale with the cantilever dimensions as $\propto\frac{1}{W{L}^{3}}$, and both are independent of the cantilever thickness. Shorter and narrower cantilevers provide a larger absolute response to analyte adsorption, but thickness has no effect in this regard. As adsorption position concerns, its effect on the responsivities remarks the important contribution of the mode shape profile. The maximum values of the factors ${c}_{M,n}\left({\xi}_{a}\right)$ and ${c}_{K,n}\left({\xi}_{a}\right)$ are of the order of a few units, and, for all modes, correspond to positions close to the free end for ${c}_{M,n}\left({\xi}_{a}\right)$ and to the clamped end for ${c}_{K,n}\left({\xi}_{a}\right)$ (Section S6, Supplementary Information). Therefore, maximum mass responsivity is obtained at adsorption positions closer to the free end, where beam displacement is maximum. Oppositely, maximum stiffness responsivity is obtained near the clamped end, where mode shape curvature is maximum.

### Resolution

The detection limits for mass and stiffness sensing of analytes upon adsorption on a beam resonator are the minimum measurable values of these magnitudes as restricted by the frequency noise $\delta{\omega}_{n}$ for each of the flexural modes of the beam [32]. If frequency noise is determined only by thermomechanical fluctuations ($\delta{\omega}_{n}^{Th}$), we can speak of fundamental detection limits because that is the lowest possible noise level at a given temperature. In such case, detection limits also define the resolution of the measurements, determining the minimum difference in mass and/or stiffness between analytes that generates discernable frequency shifts. Thus, we define the mass and stiffness resolution for the n^th^ mode of the beam, $\delta{M}_{n}$ and $\delta{K}_{n}$, respectively, by equaling each of the terms of the equation of sensitivity to the frequency stability $\delta{\omega}_{n}^{Th}$/${\omega}_{n}$, so that:

19 $$\delta{M}_{n}={2M}_{c}\frac{\delta{\omega}_{n}^{Th}}{{\omega}_{n}}$$

20 $$\delta{K}_{n}={2K}_{c}\frac{\delta{\omega}_{n}^{Th}}{{\omega}_{n}}$$

where we have omitted the coefficients ${c}_{M,n}\left({\xi}_{a}\right)$ and ${c}_{K,n}\left({\xi}_{a}\right)$ for simplicity, given that their maximum values are of a few units and we define $\delta{M}_{n}$ and $\delta{K}_{n}$ with the purpose of determining the order of magnitude of the optimal fundamental resolution for mass and stiffness. The frequency noise level $\delta{\omega}_{n}^{Th}$ given by thermomechanical fluctuations depends on the dynamic range and quality factor of each mode, and it can be written as:

21 $$\frac{\delta{\omega}_{n}^{Th}}{{\omega}_{n}}=\frac{1}{2{Q}_{n}{r}_{D,n}}$$

where ${Q}_{n}$ is the quality factor, and the dynamic range is expressed in terms of the ratio ${r}_{D,n}$ between the oscillation amplitude at the onset of nonlinearity and the amplitude corresponding to thermomechanical fluctuations. For a rectangular singly clamped beam, this ratio is given by [28]:

22 $${r}_{D,n}={a}_{n}\sqrt{\frac{{WH}^{4}}{{L}^{3}}}\sqrt{\frac{{E}_{c}^{3/2}}{{k}_{B}\stackrel{\sim}{T}{Q}_{n}^{2}B{\rho}_{c}^{1/2}}}$$

where ${k}_{B}$ is the Boltzman’s constant, $\stackrel{\sim}{T}$ is the temperature,$B$ is the measurement frequency bandwidth and ${a}_{n}$ is a coefficient that depends on the mode order, with ${a}_{1}=0.6551$, ${a}_{2}=0.5629$, ${a}_{3}=0.8956$ and ${a}_{4}=1.1963$ (Section S7, Supplementary Information). Although a singular dependence of frequency noise with quality factor has been observed in some optomechanical resonators [33], the most commonly accepted description of frequency stability as limited by thermomechanical noise is given by Eq. (21) [34–36]. Given the dependence of dynamic range with quality factor, this equation carries two relevant implications for thermomechanical noise-limited frequency stability: first, frequency stability does not depend on quality factor; second, its variation as a function of mode order remains within a small factor, given by the changes in the values of the coefficient ${a}_{n}$.

**Fig. 4:**
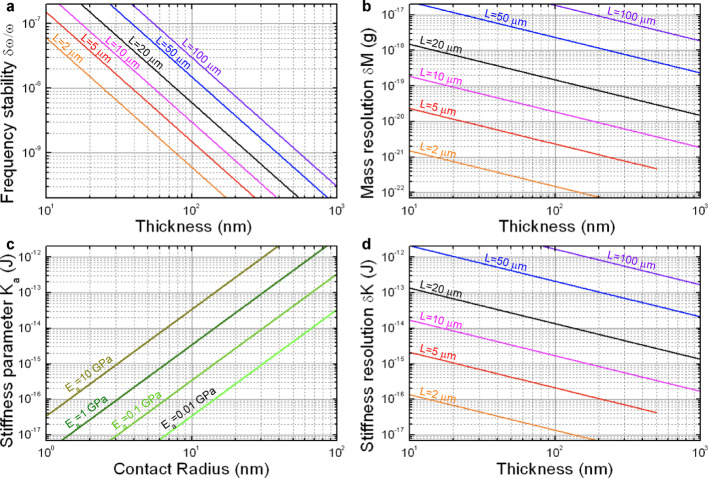
Mass and stiffness resolution as limited by thermomechanical noise for a silicon nitride beam resonator as a function of beam dimensions for the fundamental flexural mode at room temperature. **a** Frequency stability versus beam thickness for varying length, **b** Mass resolution versus beam thickness for varying length, **c** Stiffness parameter versus contact radius for different values of the Young’s modulus of the analyte as estimated by the approximation given by Eq. (15), **d** Stiffness resolution versus beam thickness for varying length. Calculations of frequency stability and resolution assume a fixed ratio $L/W=10$, a bandwidth $B=1\,\hbox{Hz}$, and a temperature$\stackrel{\sim}{T}=300\,\hbox{K}$

Figure 4a shows the resulting values for the frequency stability $\delta{\omega}_{n}^{Th}/{\omega}_{n}$ of the fundamental mode (*n* = 1) of a cantilever beam as a function of the thickness for various lengths, assuming a fixed ratio $L/W=10$, a bandwidth $B=1\,Hz$, and a temperature$\stackrel{\sim}{T}=300\,K$. The Young’s modulus and density considered for the beam are ${E}_{c}=280$ GPa and ${\rho}_{c}=3100$ kg/m^3^, typical values for silicon nitride. The graph shows that frequency noise as given by thermomechanical fluctuations decreases strongly with thickness, which is a consequence of the strongly decreasing amplitude of thermomechanical fluctuations with this dimension. It must be noted that although frequency stability can reach below 10^−9^ for cantilevers with thickness of the order of hundreds of nm, such low values are difficult to observe experimentally due to the presence of other frequency noise sources. In fact, experimental frequency stability is usually found around a factor of 10 to 100 above fundamental limits [34]. However, those other noise sources are often difficult to predict and/or to describe, so that thermomechanical noise represents a useful fundamental lower limit in the sense that any signal expected below such limit would be absolutely impossible to detect.

From the previous considerations we derive the following expressions for mass and stiffness resolution for the n^th^ flexural mode of a cantilever resonator:

23 $$\delta{M}_{n}=\frac{{M}_{c}}{{Q}_{n}{r}_{D,n}}$$

24 $$\delta{K}_{n}=\frac{{K}_{c}}{{Q}_{n}{r}_{D,n}}$$

Notably, the fundamental resolution for both mass and stiffness measurements presents the same scaling behavior with the cantilever dimensions, $\propto{{W}^{1/2}L}^{5/2}{H}^{-1}$. This behavior implies that whereas increasing width and length impairs good resolution, the effect of thickness is the opposite: thicker cantilevers result in better resolution for both mass and stiffness measurements. Figure 4b and d show respectively a calculation of the mass and stiffness resolution for the fundamental mode versus beam thickness for various values of the length at constant ratio $L/W=10$ and for $H\le W$. In the range of cantilever dimensions considered, mass resolution ranges from 10^−7^ to 10^−2^ fg, which, in terms of the application of nanomechanical spectrometry to virus detection, implies down to several orders of magnitude below the typical masses of viruses.

Regarding stiffness sensing, fundamental detection limits for the stiffness parameter ${K}_{a}$ are between 10^− 2^ and 10^3^ fJ for the considered range of dimensions. An estimation of the order of magnitude of ${K}_{a}$ for viruses can be done by using Eq. 15, as shown in Fig. 4c. Typical values of the relative contact radius ${r=R}_{C}/{R}_{0}$ of virus particles adsorbed on rigid substrates determined from previous studies are in the range 0.3–0.9, being the most frequently found value around $r=0.6$ [13, 37–42]. Given the size of viruses, this implies typical contact radii ${R}_{C}$ of the order of several tens of nm. Thus, assuming a Young’s modulus around 1 GPa and a contact radius in the 10 to 100 nm range, ${K}_{a}$ lays in the range from 1 to 10^3^ fJ. This range partly overlaps with that obtained for the detection limits, mainly for the largest cantilevers, which implies that successful application of nanomechanical spectrometry to virus stiffness sensing requires careful consideration of the resonant cantilever sensor and adsorption conditions. In particular, these estimations provide a first boundary for the cantilever dimensions and virus adsorption parameters in order to result in measurable virus stiffness: cantilevers with length below 10 μm and thickness above 100 nm as well as a virus contact radius above 10 nm are necessary in order to have a stiffness resolution at least one order of magnitude below the values estimated for the stiffness parameter ${K}_{a}$.

From the previous estimation of the resolution for the stiffness parameter ${K}_{a}$ we can derive a useful estimation of Young’s modulus resolution at constant ${R}_{0}$ and ${R}_{C}$. The definition of ${K}_{a}$ allows to relate stiffness resolution with Young’s modulus resolution by $\delta{K}_{n}=3\gamma{V}_{a}\delta{E}_{n}$. Then, using the approximation leading to Eq. 15 we can write:

25 $$\delta{E}_{n}=\frac{{K}_{c}}{3\gamma{V}_{a}{Q}_{n}{r}_{D,n}}\approx0.2935\frac{{K}_{c}}{{R}_{C}^{3}{Q}_{n}{r}_{D,n}}$$

For a silicon nitride beam resonator with $L=10\,\upmu\,\hbox{m}$, $W=1\,\upmu\,\hbox{m}$ and $H=0.5\,\upmu\hbox{m}$ we can estimate that for a contact radius ${R}_{C}$ of a few tens of nanometers, the Young’s modulus resolution is of a few MPa, which is around two orders of magnitude below the typical variations observed in the Young’s modulus of viruses as a consequence of maturation (hundreds of MPa) and also well below the observed dispersion in stiffness for viruses in the same stage of maturation [10–12]. This estimation supports the plausibility of distinguishing the maturation state, and hence the infectious potential, of viruses adsorbed at the most stiffness-sensitive positions of beam resonators. The precise extension of this highly-sensitive beam surface regions is evaluated in the next section.

Equations 23 and 24 allow to evaluate the effects of the beam material properties on mass and stiffness resolution. These magnitudes scale with the density and Young’s modulus of the beam as $\delta{M}_{n}\propto{{\rho}_{c}}^{5/4}\cdot{{E}_{c}}^{-3/4}$ and $\delta{K}_{n}\propto{{\rho}_{c}}^{1/4}\cdot{{E}_{c}}^{1/4}$, respectively. In general, the effect of density and Young’s modulus is stronger on mass than on stiffness resolution. Also, whereas density has the same effect on both, Young’s modulus has opposite contributions: a decrease in beam density improves (decreases) both mass and stiffness resolution, whereas a decrease in Young’s modulus produces an improvement on stiffness resolution accompanied by a stronger degradation (increase) of mass resolution. However, both effects are small, and the variations expected for typical materials used for the fabrication of nanomechanical resonators is smaller than those resulting from varying the beam dimensions (Section S8, Supplementary Information). For instance, if we compute the effects of moving from silicon nitride to SU-8, a polymer material with approximately a factor of 3 lower density and a factor of 100 lower Young’s modulus [43], we obtain an improvement of stiffness resolution by around a factor of 4 at the cost of a degradation of mass resolution by a factor of 8.

### Signal to noise ratio

We define SNR as the ratio of the frequency shift resulting from analyte adsorption to the frequency noise caused by thermomechanical fluctuations, which can be written in terms of mass and stiffness resolution as:

26 $$SNR=\frac{\Delta{\omega}_{n}}{\delta{\omega}_{n}^{Th}}=\frac{{K}_{a}}{\delta{K}_{n}}{c}_{K,n}\left({\xi}_{a}\right)-\frac{{M}_{a}}{\delta{M}_{n}}{c}_{M,n}\left({\xi}_{a}\right)$$

Since the total frequency shift results from the effects of the mass and stiffness of the adsorbed analyte, we define separate SNR for each effect given by:

27 $${SNR}_{M}=\frac{{M}_{a}}{\delta{M}_{n}}{c}_{M,n}\left({\xi}_{a}\right)$$

28 $${SNR}_{K}=\frac{{K}_{a}}{\delta{K}_{n}}{c}_{K,n}\left({\xi}_{a}\right)$$

Again, as the maximum values of the factors ${c}_{M,n}\left({\xi}_{a}\right)$ and ${c}_{K,n}\left({\xi}_{a}\right)$ reach a few units, the ratios $\frac{{M}_{a}}{\delta{M}_{n}}$ and $\frac{{K}_{a}}{\delta{K}_{n}}$ provide a measure of the maximum expected SNR. Given that experimental noise levels are typically a factor of 10 to 100 times that of the fundamental limit from thermomechanical fluctuations considered here [34], we consider that mass and stiffness effects are detectable when the corresponding SNR is above 100. Thus, if any of the factors $\frac{{M}_{a}}{\delta{M}_{n}}$ and $\frac{{K}_{a}}{\delta{K}_{n}}$ are below 100, then the mass and/or stiffness of the analyte is considered undetectable regardless of the adsorption position.

**Fig. 5:**
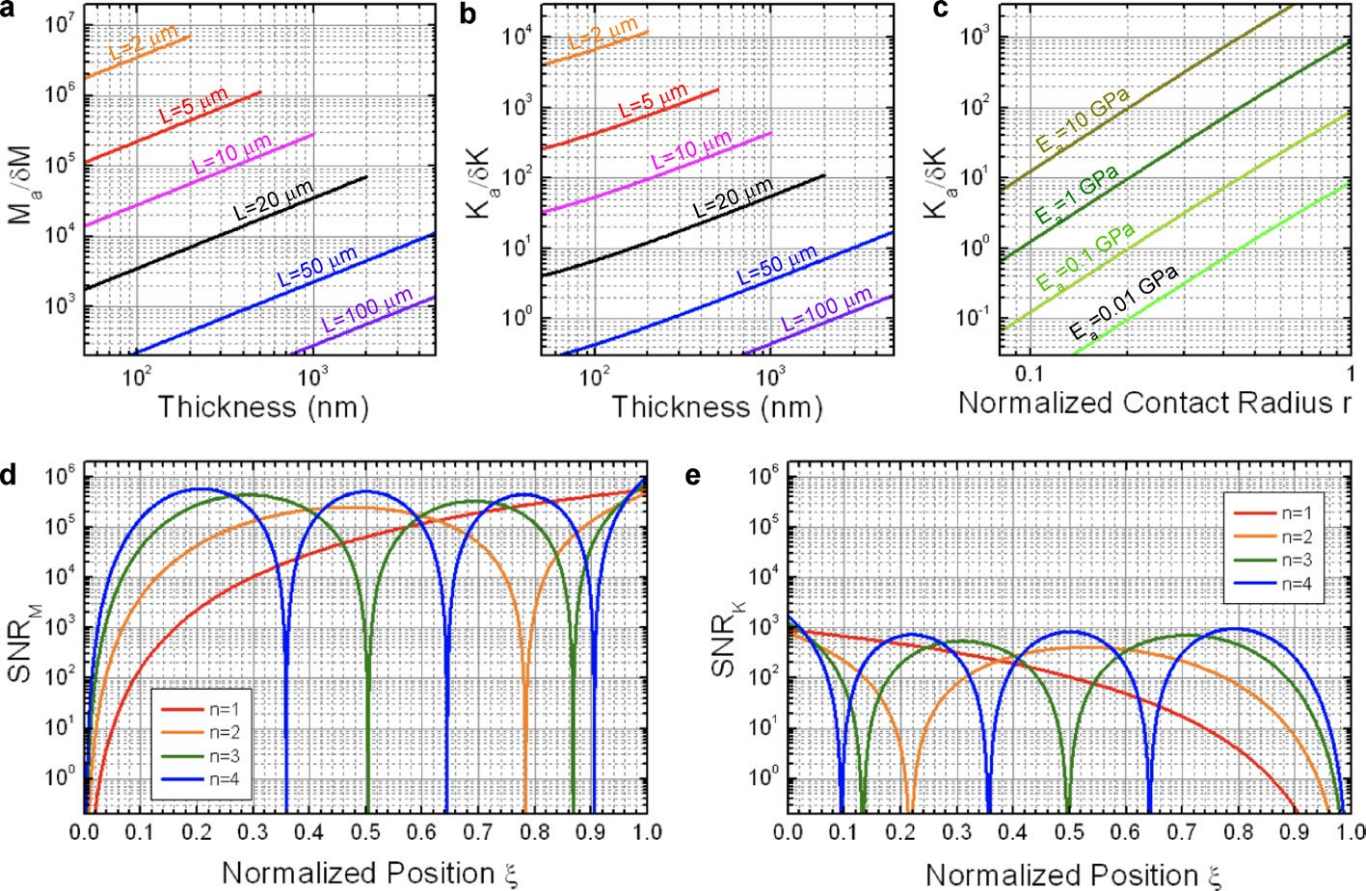
Signal to noise ratio for the adsorption of a virus SARS-CoV-2 on a silicon nitride beam resonator. **a** Ratio of virus mass to mass resolution versus beam thickness for varying length, **b** Ratio of virus stiffness parameter ${K}_{a}$ to stiffness resolution versus thickness for varying length, **c** Ratio of virus stiffness parameter ${K}_{a}$ to stiffness resolution versus normalized contact radius for varying Young’s modulus of the virus, **d** SNR for the mass contribution versus normalized adsorption position, **e** SNR for the stiffness contribution versus normalized adsorption position. The calculations in a-e assume a bandwidth $B=1\,\hbox{Hz}$ and a temperature$\stackrel{\sim}{T}=300\,\hbox{K}$. A fixed ratio$L/W=10$ is used in a-b. We consider suitable device dimensions when ${M}_{a}/\delta{M}_{n}$ and ${K}_{a}/\delta{K}_{n}$ are above 10^2^. The calculations in c-e correspond to a beam with fixed dimensions $L=10\,\upmu\hbox{m}$, $W=1\,\upmu\hbox{m}$ and $H=0.5\,\upmu\hbox{m}$. The virus parameters in a, b, d, e are ${R}_{0}=50$ nm, ${E}_{a}=1$ GPa, ${\rho}_{a}=1000$ kg/m^3^ and $r=0.6$

Figures 5a-b represent respectively the values obtained of the factors $\frac{{M}_{a}}{\delta{M}_{n}}$ and $\frac{{K}_{a}}{\delta{K}_{n}}$ for the adsorption of a virus SARS-CoV-2 on a silicon nitride beam resonator as a function of the beam thickness and length (fundamental mode only). We consider a fixed length/width ratio $L/W=10$, and plot the results for $H\le W$. We assume virus particle parameters matching those corresponding to SAR-CoV-2, with ${R}_{0}=50$ nm, ${E}_{a}=1$ GPa, ${\rho}_{a}=1000$ kg/m^3^ [44–46]. Except otherwise indicated, in this section we assume $r=0.6$. As expected from the previous analysis of mass resolution, the values of $\frac{{M}_{a}}{\delta{M}_{n}}$ are in the detectable range for all cantilever dimensions considered. However, $\frac{{K}_{a}}{\delta{K}_{n}}$ results in the detectable range only for cantilevers with length 10 μm and thickness above 200 nm or for shorter length and any thickness. The effect of varying the analyte parameters on the detectability of the analyte stiffness is shown in Fig. 5c, where $\frac{{K}_{a}}{\delta{K}_{n}}$ in the fundamental mode is represented as a function of the relative contact radius $r$ for different values of the analyte’s Young’s modulus ${E}_{a}$. This calculation is performed for a silicon nitride beam resonator with $L=10\,\upmu\hbox{m}$, $W=1\,\upmu\hbox{m}$ and $H=0.5\,\upmu\hbox{m}$. As the Young’s modulus decreases, larger values of contact radius are required in order to keep the stiffness effect into the detectable range. In particular, a Young’s modulus around 1 GPa requires a relative contact radius $r>0.4$ to result in a detectable stiffness effect.

Finally, the mass and stiffness contributions to SNR corresponding to the adsorption of a SARS-CoV-2 virus on a silicon nitride beam resonator is represented in Figs. 5d-e as a function of adsorption position for the first four flexural modes. The virus parameters are the same as in Figs. 5a-b and the fixed beam dimensions are the same as in Fig. 5c. Figure 5d shows that SNR_M_ is in the detectable range for at least three of the first four modes except for positions very close to the base (${\xi}_{a}<0.05)$. Figure 5e shows that SNR_K_ is in the detectable range for at least three of the first four modes except for positions near the free end (${\xi}_{a}>0.85)$, being this “stiffness-blind” region notably larger than the “mass-blind” portion at the base. This implies that the inverse problem method, whereby the mass, stiffness and adsorption position of the analyte are derived from the measurement of at least three different order flexural modes [23], would be applicable in this case with a sensitive area of around 80% of the total cantilever area as limited by thermomechanical noise. Relatively larger sensitive areas are obtained as cantilever length and width decrease, but that also implies a reduction of the absolute area available for adsorption. Figures 5d-e suggest a simpler though more restricted method for mass and stiffness measurements than solving the inverse problem. If adsorption position is known, for instance by optimized precise delivery of the analytes to the beam resonator [26], then the use of just the fundamental mode can provide mass and stiffness estimations from the measurement of its resonance frequency shifts. For the fundamental mode, there is a region near the base, approximately for ${\xi}_{a}<0.1$, where SNR_M_ is in the undetectable range whereas SNR_K_ is not. Conversely, there is an area near the free end, roughly for ${\xi}_{a}>0.5,$where SNR_K_ is in the undetectable range but SNR_M_ is well above. Thus, neglecting the mass term in the equation of sensitivity for the 10% of the length closer to the base can provide a straightforward determination of stiffness, whereas for the 50% of the beam length closer to the free end, it is possible to neglect the stiffness term and determine the mass from the frequency shift measurements.

## Conclusion

The equations derived in this work provide an analytical theoretical framework for the analysis of the mass and stiffness sensing performance of nanomechanical beam resonators applied for the characterization of nanoparticulate analytes. We have presented estimations based on these equations for the responsivity, resolution and SNR in order to predict which resonator dimensions and adsorption parameters result in detectable resonance frequency shifts upon the adsorption of viruses. We have shown that mass and stiffness sensing performance follow the same scaling laws and design rules, so that optimizing each of them does not compromise the other. Smaller devices optimize all sensing parameters analyzed in terms of both mass and stiffness. However, sensing optimization is not as simple as miniaturization of the resonators in all dimensions: reduced beam length and width improve sensing in regard of all performance parameters, whereas thickness behaves oppositely, particularly for resolution and SNR.

Regarding analyte adsorption, contact radius has been revealed as a critical parameter to ensure a readable stiffness signal, and adsorption positions near the beam base are expected to result in frequency shifts where the stiffness effect dominates over the mass loading contribution. In particular, a silicon nitride beam resonator with a length of 10 μm, a width of 1 μm and a thickness of 0.5 μm, which is a standard device for current state of the art microfabrication and transduction techniques, has been shown to offer a mass resolution several orders of magnitude below the mass range of viruses. Similarly, such device provides ample stiffness resolution to ensure virus stiffness sensitivity as long as the virus contact radius reaches a few tens of nanometers. Concerning the ability to sense infectivity by harnessing correlated changes in stiffness, our best estimation of Young´s modulus resolution for such device is around two orders of magnitude below the changes typically observed as a consequence of virus maturation, which supports the feasibility of an infectivity assessment mechanism founded on virus stiffness sensing.

As a consequence of the presented results, we conclude that the small size of the beam resonators required for sensitive virus detection represents the most prominent limitation as far as the development of practical applications is concerned. However, such limitation emerges not from a microfabrication or a transduction perspective, but from that of analyte delivery to the sensors. For instance, for the reference beam resonator considered above, the region corresponding to the 10% of the length closer to the base, where stiffness sensitivity is optimized, represents an area of 1 µm^2^. The first generation of analyte delivery systems that can be applied for transferring intact virus particles to beam resonators are based on the combination of electrospray ionization (ESI) with different nanoparticle beam focusing techniques, based either on aerodynamics or electrostatics. These pioneering methods have reached analyte surface density delivery rates of up to a few nanoparticles µm^− 2^ min^− 1^ when the nanoparticle concentration at the electrosprayed solution is of the order of 10^10^ nanoparticles/mL [23, 25, 26, 47]. These results allow to expect around 100 virus particles reaching the stiffness sensitive area in around 1 h for the reference beam described above. It must be considered that the number and concentration of virus particles obtained from human or environmental sampling are typically much smaller than those required for the ESI experiments developed so far [48]. Moreover, the aim of reaching infectivity assessment by stiffness sensing requires that the viruses preserve their viability during delivery to the sensors [49], which implies the implementation of methods ensuring a sufficiently soft impact of the viruses with the sensors.

In conclusion, research efforts are needed in order to maximize the efficiency of analyte delivery methods so that larger delivery rates are possible at lower initial virus particle number and concentration while preserving virus viability and minimizing the variability of contact radius. Progress in such direction would allow to exploit the suitable performance of nanomechanical sensors for rapid, untargeted and infectivity-sensitive virus detection as theoretically demonstrated here.

## Electronic supplementary material

Below is the link to the electronic supplementary material.

Supplementary Material 1

## Data Availability

The data that support the findings of this study are available from the corresponding author upon reasonable request.
