# Supplementary material for: Mass and stiffness sensing performance of nanomechanical resonators: viability of infectious virus detection
Source: Discov Nano. 2025 Jul 10;20(1):108. doi: 10.1186/s11671-025-04295-7 (PMC12246276; doi:10.1186/s11671-025-04295-7)
Supplement: Supplementary file 1 — Supplementary Material 1 [file 11671_2025_4295_MOESM1_ESM.pdf]

## Supplementary Information

# Mass and stiffness sensing performance of nanomechanical resonators: Viability of infectious virus detection

*Manuel Gómez-Moreno, Juan Molina, José J. Ruz, Óscar Malvar,*
*Javier Tamayo, Montserrat Calleja, Álvaro San Paulo**
Instituto de Micro y Nanotecnología (IMN-CNM, CSIC)
Isaac Newton 8, 28760, Tres Cantos, Madrid (Spain)
***Correspondence:** *Álvaro San Paulo* (alvaro.sanpaulo@csic.es)

## Contents

**S1. Geometry of a spherical analyte upon adsorption on a solid surface**
**S2. Rayleigh method**
**S3. Strain and elastic potential energy**
**S3.1. Strain and elastic potential energy for a flexural beam**
**S3.2. Strain and elastic potential energy of an adsorbed spherical analyte**
**S4. Finite Element Modeling**
**S5. Calculation of the strain transfer function $\gamma$**
**S5.1. Calculation of $\gamma$ from total elastic potencial energy**
**S5.2. Calculation of $\gamma$ from strain components**
**S6. Adsorption position coefficients**
**S7. Dynamic range of cantilever beam resonators**
**S8. Effect of material properties of resonators on mass and stiffness resolution**
**S9. References**

## S1. Geometry of a spherical analyte upon adsorption on a solid surface

In this section, we describe the modeling of the deformation of a solid sphere upon adsorption on a solid surface. The interaction between the adsorbate and the substrate is attributed to Van der Waals forces, which are characterized by their short-range nature.[1]

In order to simulate the deformation, we employed version 6.1 of the COMSOL Multiphysics software, utilizing the Solid Mechanics module and contact equations. Initially, a sphere in close proximity to the surface, devoid of contact, was utilized. Subsequently, a two-step stationary study was conducted. First, a volumetric force was initially applied to the sphere to establish circular contact. Then, the contact surface was fixed, and the force was eliminated, thereby releasing the stress in the sphere. It is imperative to ensure that the volume of the adsorbate remains constant throughout the process.

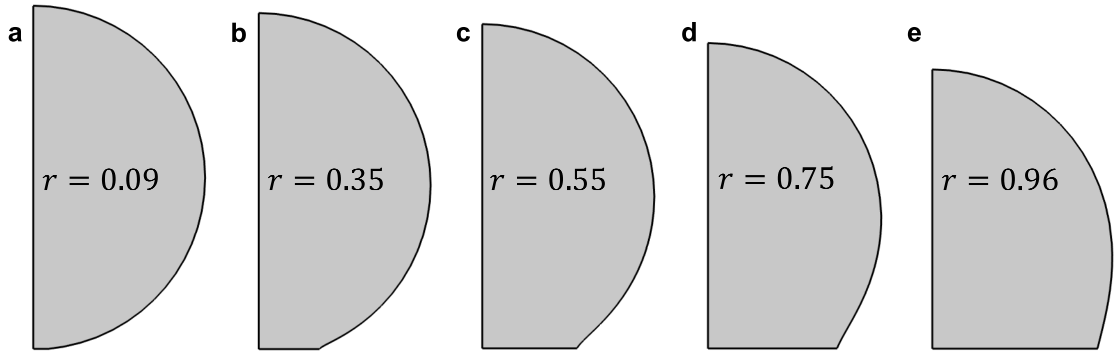


**Figure S1.1.** Deformed geometry of the sphere calculated with FEM simulations for different relative contact radii $r = R_c/R_0$. (a) $r = 0.09$, (b) $r = 0.35$, (c) $r = 0.55$, (d) $r = 0.75$, (e) $r = 0.96$.

## S2. Rayleigh method

The Rayleigh method is a technique used to calculate the eigenfrequencies $\omega_n$ of a system given its kinetic and potential energies.[2] To illustrate the method, consider a resonator of volume $V_c$ with density $\rho_c(\vec{r})$ vibrating in the n$^{\text{th}}$ eigenmode. At each vibration cycle, kinetic energy is converted into elastic potential energy according to the law of conservation of energy.[3] Consequently, this method employs the cycle-averaged energies.

The displacement vector $\overrightarrow{u_n}(\vec{r},t)$ has a temporal dependence than can be extracted by applying the method of separation of variables:

$$\overrightarrow{u_n}(\vec{r},t) = \overrightarrow{u_n}(\vec{r})\cos(\omega_n t) \qquad (S2.1)$$

The kinetic energy $T_n(t)$ and the average kinetic energy over a cycle $\langle T_n \rangle$ are given by the following expressions, where the dot denotes a time derivative[3] (note that in the following notation, when the dependence on $t$ is not explicitly written in $T_n$, we refer to it as the amplitude of $T_n(t)$):

$$T_n(t) = \frac{1}{2}\int_{V_c} \rho_c(\vec{r})\left|\dot{\overrightarrow{u_n}}(\vec{r},t)\right|^2 dV = \frac{1}{2}\omega_n^2 \int_{V_c} \rho_c(\vec{r})|\overrightarrow{u_n}(\vec{r})|^2 dV \sin^2(\omega_n t) = T_n \sin^2(\omega_n t) \quad (S2.2)$$

$$\langle T_n \rangle = \frac{\omega_n}{2\pi}\int_0^{2\pi/\omega_n} T_n(t)dt = \frac{1}{2}T_n \qquad (S2.3)$$

A normalized kinetic energy, independent of the eigenfrequency, is defined as[2]:

$$\langle \widetilde{T_n} \rangle = \frac{\langle T_n \rangle}{\omega_n^2} = \frac{1}{4}\int_{V_c} \rho_c(\vec{r})|\overrightarrow{u_n}(\vec{r})|^2 dV \qquad (S2.4)$$

The potential energy is dependent on the square of the spatial derivatives, rather than the temporal derivatives. As a result, it can be demonstrated that the temporal dependence can be extracted, and the potential energy $U_n(t)$ and its average can be calculated as follows[3]:

$$U_n(t) = U_n \cos^2(\omega_n t) \qquad (S2.5)$$

$$\langle U_n \rangle = \frac{\omega_n}{2\pi} \int_0^{2\pi/\omega_n} U_n(t) dt = \frac{1}{2} U_n \qquad (S2.6)$$

In the absence of dissipative effects, the conservation of mechanical energy can be applied. In each cycle, the maximum kinetic energy is equal to the maximum potential energy, indicating that both average energies are also the same: $\langle T_n \rangle = \langle U_n \rangle$. By employing the expression S2.4, it can be finally derived that the eigenfrequencies of the modes are:

$$\omega_n^2 = \frac{\langle U_n \rangle}{\langle \widetilde{T_n} \rangle} \qquad (S2.7)$$

## S3. Strain and elastic potential energy

In general, when a material suffers sufficiently small deformations due to the action of an external force, the dynamics of the system can be described using the theory of linear elasticity.[3] In such cases, the elastic potential energy of an infinitesimal element of volume $dV$ is given by:

$$dU = \frac{1}{2}\sigma_{ij}\varepsilon_{ij}dV \qquad (S3.1)$$

where $\varepsilon_{ij}$ are the components of the strain tensor, and $\sigma_{ij}$ are the components of the stress tensor (we use Einstein's convention of summation over repeated indexes[4]). According to Hooke's law, these tensors are related by a fourth-order tensor $C_{ijkl}$ and the Young's modulus $E$:

$$\sigma_{ij} = EC_{ijkl}\varepsilon_{kl} \qquad (S3.2)$$

If we consider a homogeneous and isotropic material, this tensor can be written as:

$$C_{ijkl} = \frac{1}{3(1-2\nu)}\delta_{ij}\delta_{kl} + \frac{1}{2(1+\nu)}\left(\delta_{ik}\delta_{jl} + \delta_{il}\delta_{jk} - \frac{2}{3}\delta_{ij}\delta_{kl}\right) \qquad (S3.3)$$

where $\nu$ is the Poisson's ratio of the material and $\delta_{ij}$ is the Kronecker delta.

These expressions are used in the subsequent subsections to calculate the energy of the cantilever and the spherical analyte.

### S3.1. Strain and elastic potential energy for a flexural beam

In this study, the focus is on singly clamped Euler-Bernoulli beams, which represent a simplification of linear elasticity.[5] These beams are three-dimensional solids with a length $L$ significantly greater than the other two dimensions, and the analysis is limited to the flexural modes of vibration. In such cases, the only non-zero components of the strain tensor are:

$$\varepsilon_{xx} = -zw''(x) \qquad (S3.4a)$$

$$\varepsilon_{yy} = \nu zw''(x) = -\nu\varepsilon_{xx} \qquad (S3.4b)$$

$$\varepsilon_{zz} = \nu zw''(x) = -\nu\varepsilon_{xx} \qquad (S3.4c)$$

where $w(x)$ corresponds to the z-component of the displacement vector $\vec{u}(\vec{r})$, and $w''(x)$ denotes the second spatial derivative. Thus, the only non-zero component of the stress tensor is:

$$\sigma_{xx} = -Ezw''(x) \qquad (S3.5)$$

The combination of these equations with equation S3.1 results in the expression for the differential potential energy of an Euler-Bernoulli singly clamped resonator:

$$dU = \frac{1}{2}Ez^2 w''(x)^2 dV \qquad (S3.6)$$

Using the definition of the normalized position $\xi = x/L$ and the established relationship between the displacement, the amplitude $A_n$ and the eigenmode $\phi_n(\xi)$, it is possible to achieve a more explicit expression of the energy of the n$^{th}$ flexural mode:

$$dU_n = \frac{1}{2}E\frac{z^2}{L^4}A_n^2 \phi_n''(\xi)^2 dV \qquad (S3.7)$$

Finally, the potential energy of the cantilever is calculated by integrating the previous expression over the volume of the resonator.

$$U_n = \frac{1}{2}\frac{E}{L^4}A_n^2 \int_{-\frac{W}{2}}^{\frac{W}{2}} dy \int_{-\frac{H}{2}}^{\frac{H}{2}} z^2 dz \int_0^1 \phi_n''(\xi)^2 L d\xi = \frac{1}{2}\frac{EV}{12}\frac{H^2}{L^4}A_n^2 \int_0^1 \phi_n''(\xi)^2 d\xi \qquad (S3.8)$$

**S3.2. Strain and elastic potential energy of an adsorbed spherical analyte**

In the case of a spherical analyte with a deformed geometry as described in section S1, the strain distribution is quite complex and there is no general analytical solution. However, it can be assumed that the strain inside the adsorbate $\varepsilon_{ij}^a$ is a consequence of the transference of strain from the cantilever, and therefore, the components of the strain of the analyte can be expressed as a linear combination of the in-plane components of the strain of the cantilever $\varepsilon_{mq}^c$ at the point of adsorption $(x_0, y_0, z_0)$.[6]

$$\varepsilon_{ij}^{a}(x,y,z) = a_{ijmq}(x,y,z)\varepsilon_{mq}^{c}(x_0,y_0,z_0) \qquad (S3.9)$$

where $a_{ijmq}(x,y,z)$ is a function that couples the strain in the cantilever and the adsorbate, and $m,q \in \{x,y\}$. If we combine this equation with the equation S3.1 of the potential energy applied to the analyte, we obtain a new expression for the potential energy:

$$dU_a = \frac{1}{2}E_a\varepsilon_{mq}^{c}(x_0,y_0,z_0)\varepsilon_{rs}^{c}(x_0,y_0,z_0)C_{ijkl}a_{ijmq}a_{klrs}dV \qquad (S3.10)$$

We can define the differential strain transfer function as[6]:

$$d\gamma_{mqrs} = C_{ijkl}a_{ijmq}a_{klrs}dV/V_a \qquad (S3.11)$$

where $V_a$ is the volume of the adsorbate. Knowing that the only non-zero components of $\varepsilon_{mq}^{c}$ are $\varepsilon_{xx}^{c}$ and $\varepsilon_{yy}^{c}$, and that they are proportional such that $\varepsilon_{yy}^{c} = -\nu_c\varepsilon_{xx}^{c}$ (eq. 3.4b), we reach:

$$dU_a = \frac{1}{2}E_aV_a\varepsilon_{xx}^{c}(x_0,y_0,z_0)^2 d\gamma \qquad (S3.12)$$

where we defined $d\gamma = d\gamma_{xxxx} - 2\nu_c d\gamma_{xxyy} + \nu_c^2 d\gamma_{yyyy}$.

If we consider that the analyte is small enough compared to the cantilever so that the curvature $\phi_n''(\xi)$ is constant along the contact area, then we can integrate the equation S3.12 to obtain the elastic potential energy of the analyte:

$$U_a = \frac{1}{2}\frac{E_aV_a}{4}\frac{H^2}{L^4}\gamma A_n^2\phi_n''(\xi_a)^2 \qquad (S3.13)$$

where $\gamma$ is the strain transfer function, and $A_n$ is the amplitude of oscillation of the n$^{th}$ flexural mode and it relates the eigenmode and the displacement: $w_n(x) = A_n\phi_n(x)$.

## S4. Finite Element Modeling

Finite Element simulations were performed to analyze the effect of a solid spherical analyte with a deformed geometry (see section S1) on the surface of a singly clamped Euler-Bernoulli beam. Version 6.1 of the COMSOL Multiphysics software was utilized, and the model was constructed using the Solid Mechanics module with Eigenfrequency studies. The mesh was refined until a 99.9% of convergence on the solution was achieved.

The parameters of the simulations, unless explicitly stated otherwise, are as follows:

| Parameter | Value | Definition |
|---|---|---|
| $L$ | 20 µm | Length of the cantilever |
| $W$ | 2 µm | Width of the cantilever |
| $H$ | 1 µm | Thickness of the cantilever |
| $E_c$ | 280 GPa | Young's modulus of the cantilever ($Si_3N_4$) |
| $\rho_c$ | 3100 kg/m$^3$ | Density of the cantilever ($Si_3N_4$) |
| $\nu_c$ | 0.23 | Poisson's ratio of the cantilever[7] |
| $\xi_0$ | 0.1 | Normalized adsorption position |
| $R_0$ | 50 nm | Radius of the adsorbate |
| $E_a$ | 1 GPa | Young's modulus of the adsorbate |
| $\rho_a$ | 1000 kg/m$^3$ | Density of the adsorbate |
| $\nu_a$ | 0.25 | Poisson's ratio of the adsorbate |

**Table S4.1.** Parameters of the FEM simulations.

## S5. Calculation of integrated strain transfer function $\gamma$

### S5.1. Calculation of $\gamma$ from total elastic potencial energy

The aim of this section is to derive an analytical approximation for the strain transfer function $\gamma$ utilizing the potential energy of the adsorbate calculated with FEM simulations (see section S4). Two normalized parameters of interest are defined for the simulation (Fig. 5.1a): the normalized contact radius, defined as $r = R_c/R_0$, and the normalized size of the analyte, represented by $\eta = R_0/H$.

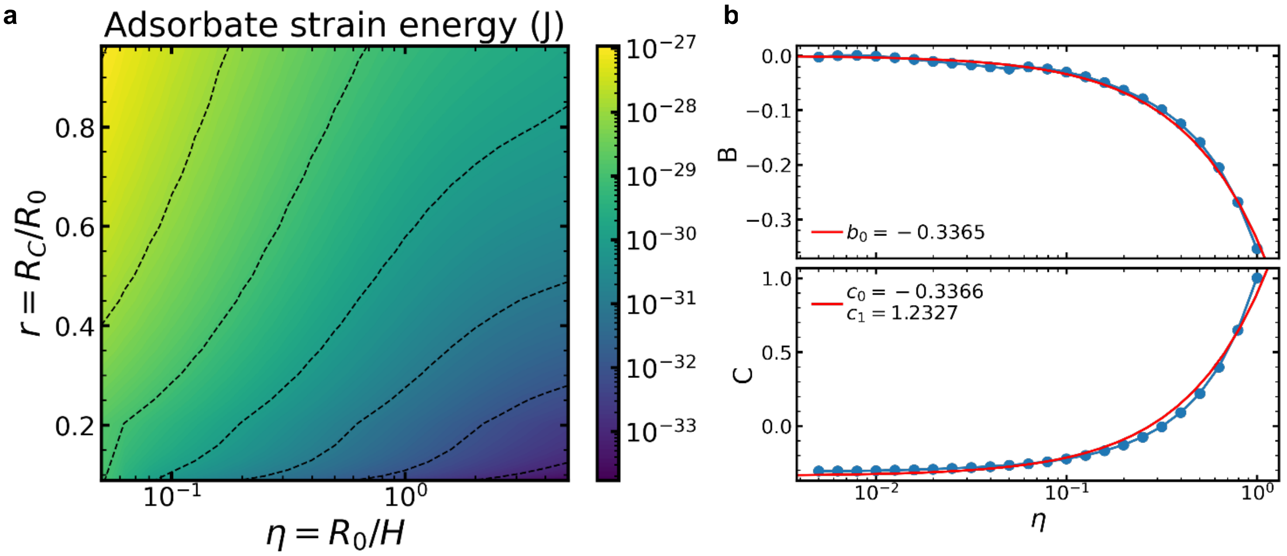


**Figure S5.1.** (a) Adsorbate elastic potential energy of the first flexural mode calculated with FEM simulations for an analyte with $E_a = 1GPa$ at adsorption position $\xi_a = 0.1$ for different contact radii and relative sizes. (b) Fitting of the parameters that depend on the relative size. Blue dots represent the data obtained from the simulation and the red line indicates the polynomial fitting.

Using equation S3.13 we are able to obtain the values of $\gamma$ for every energy. Subsequently, we fit these values to a two-variable function. Initially, we group all the calculated $\gamma$ with the same value of $\eta$ and refer to this set as $\gamma_{\eta_i}$. Then, we fit each one of them to a function $\gamma_{\eta_i} = Ar^3(1 + B + Cr)$, where $A$, $B$ and $C$ are the fitting parameters. A common value of $A = 0.2711$ is found for all $\gamma_{\eta_i}$, while $B$ and $C$ depend on $\eta$. Subsequently, the values of these two parameters are grouped and fitted to two different polynomials (Fig. S5.1b): $B = b_0\eta$ and $C = c_0 + c_1\eta$. The final dependence of the strain transfer function with the contact radius and the relative size is:

$$\gamma \approx 0.2711r^3(1 - 0.3365\eta - 0.3366r + 1.2327\eta r) \qquad (S5.1)$$

**S5.2. Calculation of $\gamma$ from strain components**

In this section, the objective is to calculate an explicit expression of the strain transfer function $\gamma$ in terms of the strain components $\varepsilon_{ij}^{a}$ rather than the energy, as seen in section S5.1. The aim is to solve equation S3.11. However, the components of the tensor $a_{ijkl}$ are unknown. These components can be calculated them using equation S3.9 with the strain components of the cantilever (equation S3.4):

$$\widehat{\varepsilon_{ij}} = \left(a_{ijxx} - \nu_c a_{ijyy}\right) \qquad (S5.2)$$

where $\nu_c$ is the Poisson's ratio of the cantilever and we defined the normalized strain $\widehat{\varepsilon_{ij}} \equiv \varepsilon_{ij}^{a}/\varepsilon_{xx}^{c}(\overrightarrow{r_0})$. The strain tensor is a symmetric tensor, and consequently, $a_{ijkl}$ has the so-called major and minor symmetries of a fourth order tensor,[8] i.e., $a_{ijkl} = a_{jikl} = a_{ijlk} = a_{klij}$. Therefore, there are only six independent components of equation S5.2:

$$\widehat{\varepsilon_{xx}} = \left(a_{xxxx} - \nu_c a_{xxyy}\right) \qquad (S5.3a)$$
$$\widehat{\varepsilon_{xy}} = \left(a_{xxxy} - \nu_c a_{xyyy}\right) \qquad (S5.3b)$$
$$\widehat{\varepsilon_{xz}} = \left(a_{xxxz} - \nu_c a_{xzyy}\right) \qquad (S5.3c)$$
$$\widehat{\varepsilon_{yy}} = \left(a_{xxyy} - \nu_c a_{yyyy}\right) \qquad (S5.3d)$$
$$\widehat{\varepsilon_{yz}} = \left(a_{xxyz} - \nu_c a_{yyyz}\right) \qquad (S5.3e)$$
$$\widehat{\varepsilon_{zz}} = \left(a_{xxzz} - \nu_c a_{yyzz}\right) \qquad (S5.3f)$$

However, FEM simulations (see section S4) show that $\varepsilon_{xy}^{a}$ and $\varepsilon_{yz}^{a}$ are always zero, thereby indicating that coefficients $a_{xxxy}$, $a_{xyyy}$, $a_{xxyz}$ and $a_{yyyz}$ must be zero. It is noteworthy that half of the components appear multiplied by the Poisson's ratio of the cantilever. For this reason, an additional set of FEM simulations was conducted with $\nu_c = 0$, and the results were compared with the initial set. First, the component $\varepsilon_{yy}^{a}$ completely cancelled when the Poisson's ratio was zero, indicating $a_{xxyy} = 0$ (eq. S5.3d). Second, the component $\varepsilon_{xz}^{a}$ remained unchanged regardless of the value of $\nu_c$, leading to $a_{xzyy} = 0$ (eq. S5.3c). Consequently, only five non-zero components of the tensor $a_{ijkl}$ remain, and therefore, there are only four equations:

$$\widehat{\varepsilon_{xx}} = a_{xxxx} \qquad (S5.4a)$$

$$\widehat{\varepsilon_{xz}} = a_{xxxz} \tag{S5.4b}$$

$$\widehat{\varepsilon_{yy}} = -\nu_c a_{yyyy} \tag{S5.4c}$$

$$\widehat{\varepsilon_{zz}} = \left(a_{xxzz} - \nu_c a_{yyzz}\right) \tag{S5.4d}$$

The next step is to calculate the tensor $C_{ijkl}$ with the Poisson's ratio of the analyte. We can notice from equation S3.3 that there are just three cases in which the components are non-zero, which are:

$$C_{iiii} = \frac{1-\nu_a}{(1+\nu_a)(1-2\nu_a)} \equiv C_0 \tag{S5.5a}$$

$$C_{iijj,\ i\neq j} = \frac{\nu_a}{(1+\nu_a)(1-2\nu_a)} \equiv C_1 \tag{S5.5b}$$

$$C_{ijij,\ i\neq j} = \frac{1}{2(1+\nu_a)} \equiv C_2 \tag{S5.5c}$$

Putting all together in equation S3.11 leads to:

$$d\gamma = \left[C_0\left(\widehat{\varepsilon_{xx}}^2 + \widehat{\varepsilon_{yy}}^2 + \widehat{\varepsilon_{zz}}^2\right) + 2C_1\left(\widehat{\varepsilon_{xx}}\widehat{\varepsilon_{yy}} + \widehat{\varepsilon_{xx}}\widehat{\varepsilon_{zz}} + \widehat{\varepsilon_{yy}}\widehat{\varepsilon_{zz}}\right) + 4C_2\widehat{\varepsilon_{xz}}^2\right]dV/V_a \tag{S5.6}$$

We can now define the in-plane and out-of-plane components ($d\gamma_{\parallel}$ and $d\gamma_{\perp}$) combining the contributions without and with z-component, respectively. Notice that $d\gamma = d\gamma_{\parallel} + d\gamma_{\perp}$.

$$\gamma_{\parallel} = \left[C_0\left(\widehat{\varepsilon_{xx}}^2 + \widehat{\varepsilon_{yy}}^2\right) + 2C_1\widehat{\varepsilon_{xx}}\widehat{\varepsilon_{yy}}\right]dV/V_a \tag{S5.7a}$$

$$\gamma_{\perp} = \left[C_0\widehat{\varepsilon_{zz}}^2 + 2C_1\left(\widehat{\varepsilon_{xx}}\widehat{\varepsilon_{zz}} + \widehat{\varepsilon_{yy}}\widehat{\varepsilon_{zz}}\right) + 4C_2\widehat{\varepsilon_{xz}}^2\right]dV/V_a \tag{S5.7b}$$

Figure S5.2 shows a calculation of the in-plane $\gamma_{\parallel}$ and out-of-plane $\gamma_{\perp}$ components of the strain transfer function obtained from the FEM calculations of the corresponding strain components. These components satisfy $\gamma = \gamma_{\parallel} + \gamma_{\perp}$. For both components, the dominant $\propto r^3$ behavior is evident, although more important deviations are observed for the out-of-plane component $\gamma_{\perp}$, particularly at larger $r$ (Figure S5.2a). On the other hand, $\gamma_{\perp}$ also shows a stronger dependence on $\eta$, specially at larger values of $r$, whereas the effect of $\eta$ on the in-plane component $\gamma_{\parallel}$ is much weaker at any value of $r$ (Figure S5.2b). As discussed in the main text, the FEM calculation of the different strain components in the analyte show that the in-plane strain components $\varepsilon_{xx}$ and $\varepsilon_{yy}$ are dominated by a

strain release behavior, whereas in the out-of-plane components $\varepsilon_{zz}$ and $\varepsilon_{xz}$, border effects prevail (Figure 2 of the main text). Thus, neglecting the terms in $\eta$ in the expression obtained for $\gamma$, and thus for the calculation of $K_a$, can be interpreted as partly neglecting the contribution from border effects, or considering analyte stiffness dominated by strain release, given that the effect of $\eta$ is stronger in the out-of-plane component of $\gamma$.

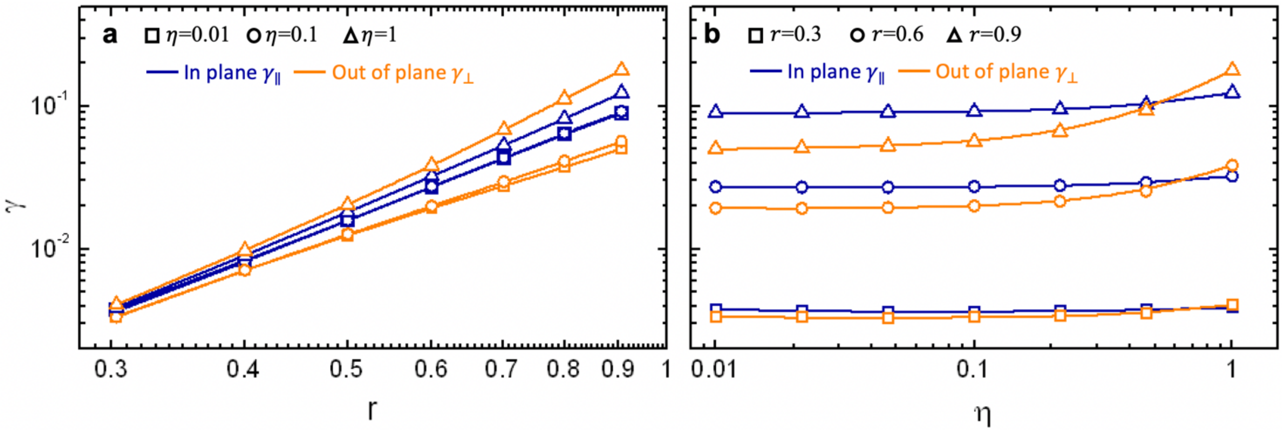


**Figure S5.2.** Calculation of in-plane and out-of-plane components of the strain transfer function $\gamma$. (a) Separated in-plane and out-of-plane contributions to $\gamma$ as a function of $r$ for $\eta$ = 0.01, $\eta$ = 0.1 and $\eta$ = 1. (d) Separated in-plane and out-of-plane contributions to $\gamma$ as a function of $\eta$ for $r$ = 0.3, $r$ = 0.6 and $r$ = 0.9.

## S6. Adsorption position coefficients

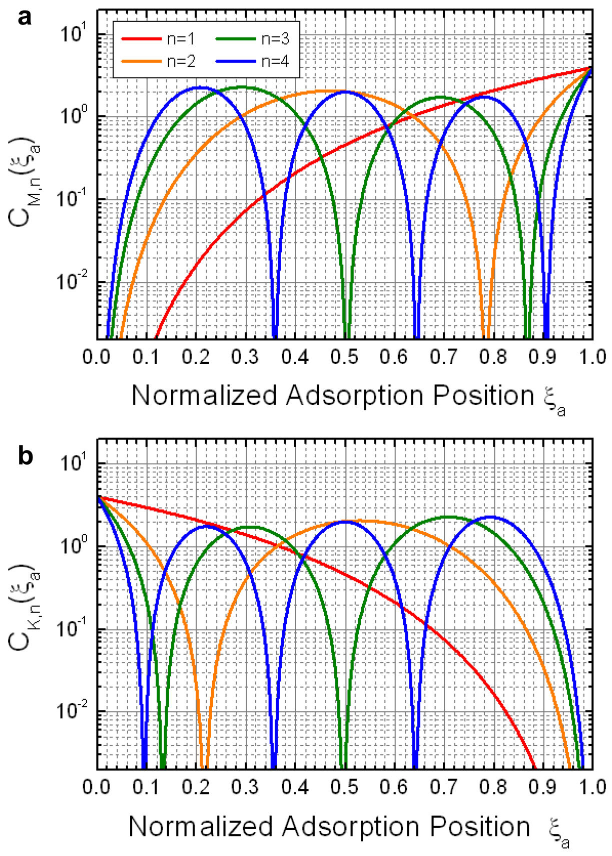


**Figure S6.1.** Coefficients of analyte adsorption position. (a) Coefficient for the mass contribution as a function of the normalized adsorption position along the longitudinal coordinate. (b) Coefficient for the stiffness contribution as a function of the normalized adsorption position along the longitudinal coordinate.

## S7. Dynamic range of cantilever beam resonators

The dynamic range of flexural modes is determined by the ratio $r_{D,n}$ between the oscillation amplitude at the onset of nonlinearity and that corresponding to thermomechanical fluctuations. In a previous work, an expression for $r_{D,n}$ was calculated for the fundamental flexural mode.[9] This can be generalized to any flexural mode of order $n$ as:

$$r_{D,n} = \frac{\left(10^{1/10}-1\right)^{1/4}}{\sqrt{6}} \sqrt{\frac{L^2 m_n \omega_n^3}{k_B \tilde{T} B |\alpha_n^{NL}| Q^2}} \qquad (S7.1)$$

where the effective mass $m_n$, the angular resonance frequency $\omega_n$ and the nonlinear coefficient $\alpha_n^{NL}$ depend on the mode order according to:

$$m_n = \rho_c LWH \int_0^1 \phi_n(\xi)^2 d\xi \qquad (S7.2)$$

$$\omega_n = \beta_n^2 \frac{H}{L^2} \sqrt{\frac{E_c}{12\rho_c}} \qquad (S7.3)$$

$$\alpha_n^{NL} = \frac{2\int_0^1 [\phi_n'(\xi)\phi_n''(\xi)]^2 d\xi}{\int_0^1 \phi_n''(\xi)^2 d\xi} - \frac{2}{3} \frac{\int_0^1 \left[\int_0^\xi \phi_n'(\zeta)^2 d\zeta\right]^2 d\xi}{\int_0^1 \phi_n(\xi)^2 d\xi} \qquad (S7.4)$$

being $\beta_n = \{1.875, 4.694, 7.855\}$ for $n = \{1,2,3\}$ and $\beta_n = (2n-1)\pi/2$ for $n \geq 4$.[10] Thus, considering the mode shape functions:

$$\phi_n(\xi) = \cosh(\beta_n \xi) - \cos(\beta_n \xi) + \frac{\cos(\beta_n) + \cosh(\beta_n)}{\sin(\beta_n) + \sinh(\beta_n)} [\sin(\beta_n \xi) - \sinh(\beta_n \xi)] \qquad (S7.5)$$

and solving numerically the integrals from expressions S7.2 and S7.4, we obtain:

$$r_{D,n} = a_n \sqrt{\frac{WH^4}{L^3}} \sqrt{\frac{E_c^{3/2}}{k_B \tilde{T} Q_n^2 B \rho_c^{1/2}}} \qquad (S7.6)$$

with $a_n = \{0.6551, 0.5629, 0.8956, 1.1963\}$ for $n = \{1,2,3,4\}$.

## S8. Effect of material properties of resonators on mass and stiffness resolution

Figure S8.1 show a calculation of mass and stiffness resolution (fundamental mode) vs. beam thickness for various values of the length and for different materials at constant ratio $L/W = 10$, bandwidth $B = 1\ Hz$, temperature $\tilde{T} = 300\ K$, and for $H \leq W$. The material properties for the cases considered are: Silicon, $\rho_c$= 2330 kg/m$^3$, $E_c$=130 GPa; Gallium Arsenide, $\rho_c$= 5320 kg/m$^3$, $E_c$=85.5 GPa; SU-8, $\rho_c$= 1190 kg/m$^3$, $E_c$=4 GPa. The calculations show that these variations in material properties produce variations in mass and stiffness resolution that remain within one order of magnitude. This is a consequence of the scaling behavior of these magnitudes with the material properties, as discussed in the main text.

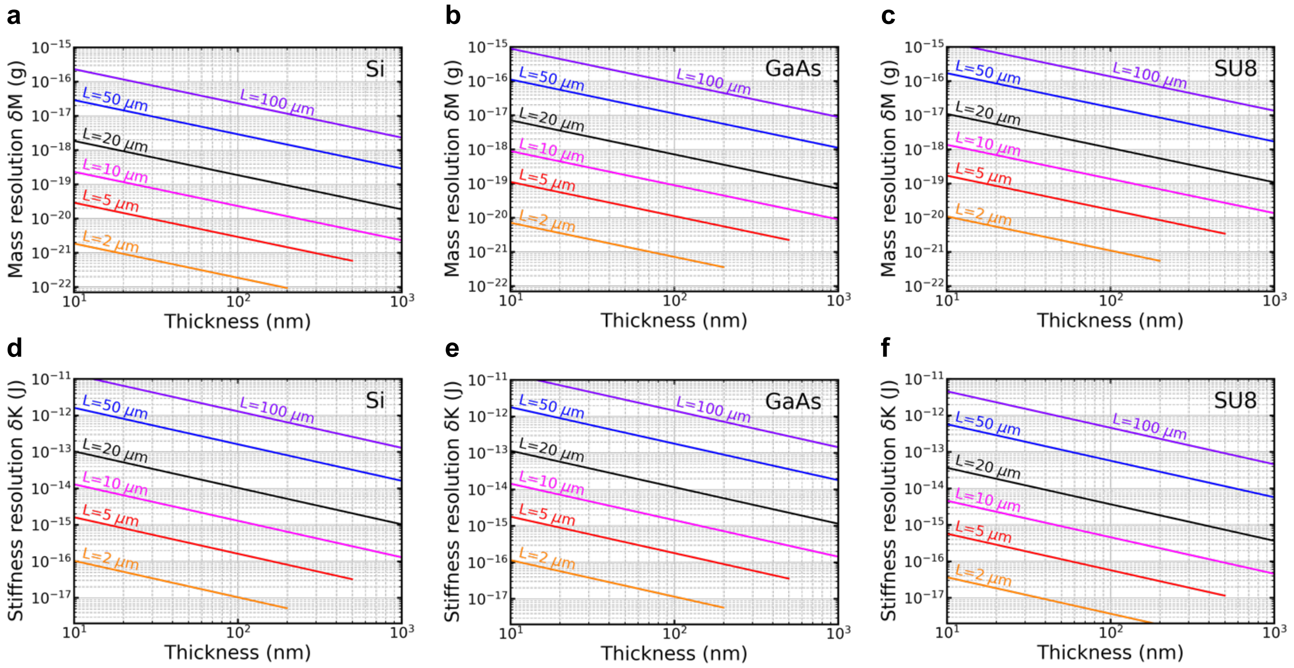


**Figure S8.** Mass and stiffness resolution as limited by thermomechanical noise for beam resonators made of different materials as a function of beam dimensions for the fundamental flexural mode at room temperature. (a-c) Mass resolution vs. beam thickness for varying length for beam material properties corresponding to that of Silicon (a), Gallium Arsenide (b) and SU-8 (c). (d-f) Stiffness resolution vs. beam thickness for varying length for beam material properties corresponding to that of Silicon (d), Gallium Arsenide (e) and SU-8 (f).

## S9. References


1. Landau, L. D., Lifshitz, E. M. & Pitaevskii, L. P. *Electrodynamics of Continuous Media*. vol. 8 (1984).
2. Leissa, A. W. The historical bases of the Rayleigh and Ritz methods. *J Sound Vib.* **287**, 961–978 (2005).
3. Landau, L. D., Lifshitz, E. M., Pitaevskii, L. P. & Kosevich, A. M. *Theory of Elasticity*. vol. 7 (Elsevier Science, 1986).
4. Einstein, A. Die Grundlagen der allgemeinen. *Relativitats- teorie, Annale del Physic* **49**, 769 (1916).
5. Timoshenko, S. *History of Strength of Materials: With a Brief Account of the History of Theory of Elasticity and Theory of Structures*. (McGraw-Hill, New York, 1983).
6. Ruz, J. J., Malvar, O., Gil-Santos, E., Calleja, M. & Tamayo, J. Effect of particle adsorption on the eigenfrequencies of nano-mechanical resonators. *J. Appl. Phys.* **128**, 104503 (2020).
7. Ruz, J. J., Tamayo, J., Pini, V., Kosaka, P. M. & Calleja, M. Physics of nanomechanical spectrometry of viruses. *Sci Rep* **4**, (2014).
8. Itin, Y. & Hehl, F. W. The constitutive tensor of linear elasticity: Its decompositions, Cauchy relations, null Lagrangians, and wave propagation. *J Math Phys* **54**, 42903 (2013).
9. Molina, J., Escobar J. E., Ramos, D., Gil-Santos, E., Ruz, J. J., Tamayo, J., San Paulo, A., Calleja, M., High Dynamic Range Nanowire Resonators. *Nano Lett* **21**, 6617–6624 (2021).
10. Schmid, S., Villanueva, L. G. & Roukes, M. L. *Fundamentals of Nanomechanical Resonators*. vol. 49 (Springer, Berlin, 2016).
